# Imaging atomic-level random walk of a point defect in graphene

**DOI:** 10.1038/ncomms4991

**Published:** 2014-05-29

**Authors:** Jani Kotakoski, Clemens Mangler, Jannik C. Meyer

**Affiliations:** 1 Faculty of Physics, University of Vienna, Boltzmanngasse 5, A-1090 Vienna, Austria; 2 Department of Physics, University of Helsinki, PO Box 43, FI-00014 Helsinki, Finland

## Abstract

Deviations from the perfect atomic arrangements in crystals play an important role in affecting their properties. Similarly, diffusion of such deviations is behind many microstructural changes in solids. However, observation of point defect diffusion is hindered both by the difficulties related to direct imaging of non-periodic structures and by the timescales involved in the diffusion process. Here, instead of imaging thermal diffusion, we stimulate and follow the migration of a divacancy through graphene lattice using a scanning transmission electron microscope operated at 60 kV. The beam-activated process happens on a timescale that allows us to capture a significant part of the structural transformations and trajectory of the defect. The low voltage combined with ultra-high vacuum conditions ensure that the defect remains stable over long image sequences, which allows us for the first time to directly follow the diffusion of a point defect in a crystalline material.

## Abstract (web summary)

Observing the movement of defects through a crystal lattice in real time presents significant difficulties. Here, the authors use an ultra-high vacuum and low-voltage scanning transmission microscope setup to observe the migration of a divacancy defect in real time through graphene.

## Graphical abstract

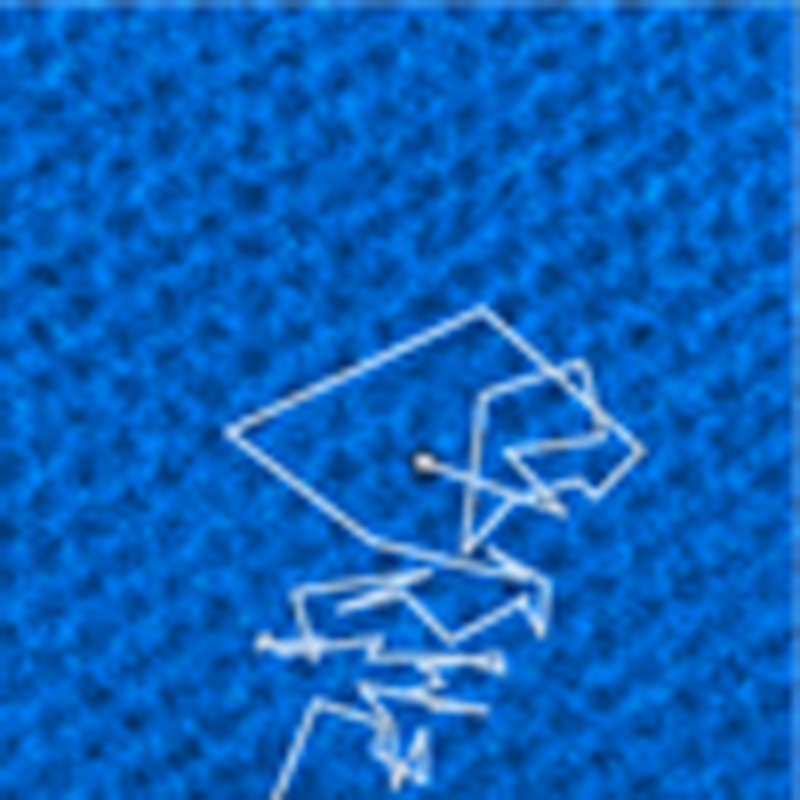

High-resolution electron microscopy has recently exposed the atomic structure of two-dimensional (2D) materials such as graphene^1^,^2^, hexagonal boron nitride^3^,^4^ and transition metal dichalcogenide^5^ monolayers, and a 2D silica glass structure^6^ for direct observation. Modern imaging techniques are also able to directly discern between different chemical elements in these structures, even for atoms that are neighbours in the periodic table^7^,^8^,^9^,^10^. In addition to structural and chemical analysis, electron microscopy has also led to advances in the understanding of dynamical, mostly beam-driven, processes in graphene and similar materials. For example, imaging with 80 keV electrons has been shown to excite structural changes in pristine graphene^2^, and at its point defects^11^,^12^,^13^,^14^, grain boundaries^15^ and dislocations^16^,^17^, whereas 60 keV imaging has revealed dynamics of a six-atomic Si cluster^18^ in graphene. However, beam-induced knock-on damage with less than ideal vacuum conditions have until the current state-of-the-art instruments prevented direct observation of point defect migration in pristine graphene over long image sequences. Here, using 60 keV imaging, we reveal at the atomic resolution a random walk performed by a defect in an otherwise perfect graphene crystal.

## Results

### Image sequences of divacancy migration

**Figure 1:**
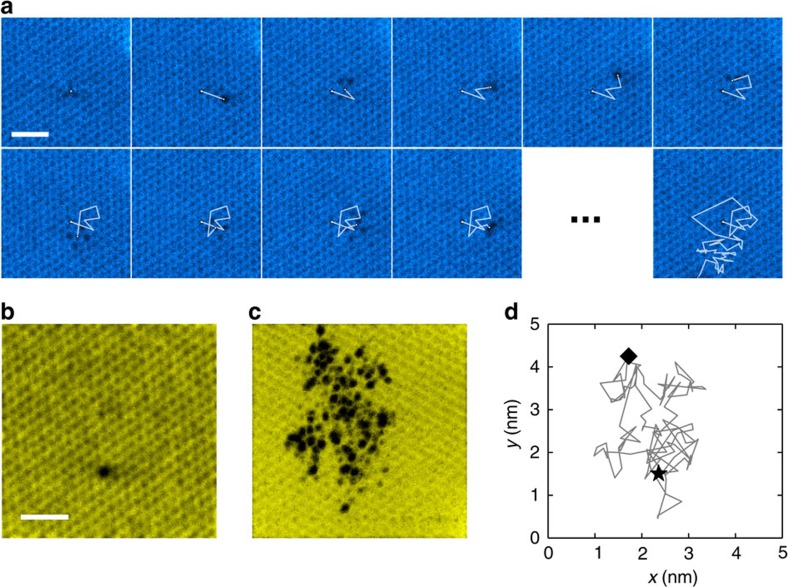
Travelling divacancy in graphene. (**a**) Ten consecutive frames and the final frame from one image sequence showing the movement of the defect through the lattice. (**b**) First frame of another image sequence. (**c**) ‘Superposition’ of all of the frames from the second sequence highlighting the trace of the defect by showing the minimum intensity from the sequence at every pixel. (**d**) Actual trajectory of the defect in the second sequence, determined by locating the approximate middle point of the defect in every frame. Only those images where the location of the defect was clearly identifiable have been included. The start position is marked with a black star and the last location with a diamond. All scale bars are 1 nm.

Our data consist of two image sequences of a divacancy defect in monolayer graphene (with 57 and 143 frames, recorded over about 5 min 32 s and 12 min 59 s, respectively), obtained with a Nion UltraSTEM 100 (ref. 19) electron microscope operated at 60 kV. Owing to the low voltage and the ultra-high vacuum conditions (1.3 × 10^−9^ mbar at the sample), the divacancy in graphene is extraordinarily stable: it neither converts into higher-order vacancies nor traps carbon and converts back to a pristine lattice, for long sequences of images. However, the defect rapidly moves via beam-driven bond rotations during observation, and constantly changes shape between four different configurations, which have also been identified in earlier images^20^. As a result, the defect performs a random walk through the lattice. A sequence of 10 consecutive frames and a final frame of one image sequence is presented in Fig. 1a as an example. Figure 1b shows the first frame from another image sequence. Figure 1c is a ‘superposition’ of all frames highlighting the trace of the defect (by showing the minimum of intensity from the sequence at every pixel), and Fig. 1d depicts the defect trajectory from the same sequence, obtained by locating the center of the divacancy in every frame. Complete sequences are provided as Supplementary Movie 1 for the first image sequence and as Supplementary Movie 2 for the second image sequence. Non-treated versions of all images are provided as Supplementary Movies 3 and 4 for the first and the second sequence, respectively.

### Atomic configurations of the divacancy

**Figure 2:**
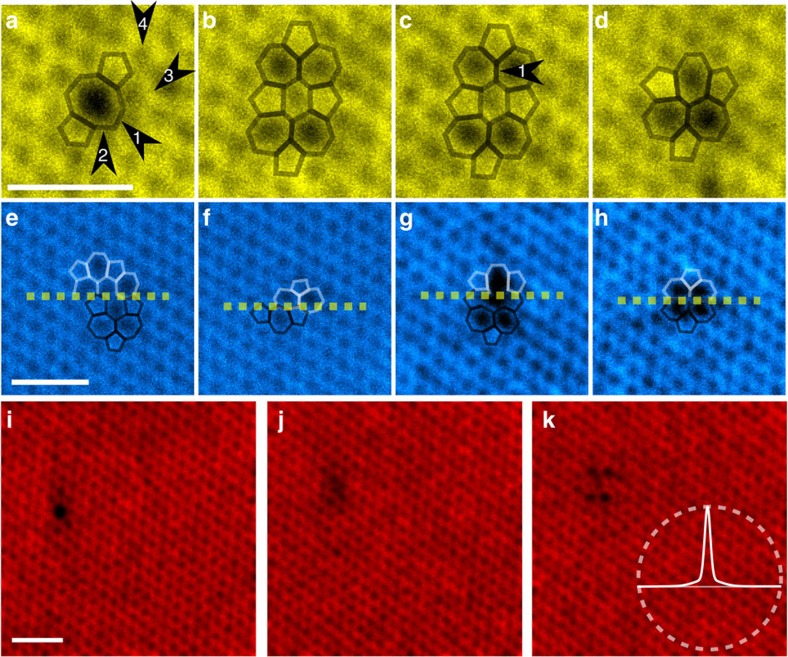
Example exposures of the defect. (**a**–**d**) Four consecutive frames from the second image sequence. The bonds associated with the defects are highlighted with an overlay. The structure has undergone at least four bond rotations between panels **a** and **b** and one between **c** and **d**, as marked with the black arrows. (**e**–**h**) Examples of image scans where the structure changed while scanning exactly at the location of the defect. White and black overlays mark the structure of the defect before and after the change, respectively. (**i**–**k**) Three consecutive frames from the second image sequence. The defect appears in the V_2_(585) configuration in panel **i**, but disappears for the duration of the next scan resulting in panel **j**, before appearing again in panel **k**, in the V_2_(5555-6-7777) configuration. The darker area within panel **j** presumably corresponds to the area where the defect is located, although it avoids detection (locations of all atoms belonging to the pristine lattice can be identified). In panel **k**, a circle with radius of 1.5 nm is drawn for scale with the experimentally obtained probe shape. In these panels the complete field of view is shown. The scale bars are 1 nm.

Figure 2a–d shows the defect in three different configurations: V_2_ (585) in panel a, V_2_ (5555-6-7777) in panels b and c and V_2_ (555-777) in panel d; (in this notation, the carbon rings contained within the defects are listed, when possible, along the longest axis through the defect). Owing to the symmetry of the lattice, V_2_(555-777) can appear with two distinct orientations, whereas the other two have three possible orientations. Throughout the data, these defects have the following probabilities to occur: 50.3% for V_2_ (585), 14.1% for V_2_ (555-777) and 18.8% for V_2_(5555-6-7777). In addition, in three frames (1.8%) the defect appears in the 2 × (57) configuration^21^. The rest (14.7%) of the frames contain either unclear structures or combinations of two or more of the above mentioned configurations. Examples of these are shown in Fig. 2e–h. In two frames the defect has completely eluded detection although it is visible both in the previous and the following frames (an example is presented in Fig. 2i–k).

### Defect transformations

As mentioned, <15% of the scan images showed any indications of the structure changing during the scan. This is surprising, because the consecutive scans nevertheless revealed the defect in other locations and often in another configuration. It is almost as if the scans would correspond to photographs taken in a busy but dark room, only momentarily illuminated by the flash of the camera freezing the moment in time. However, it is well understood that the bond rotations, which are responsible for the observed structural changes, are associated with an energy barrier of 5–10 eV^22^ and can thus not be driven by thermal activation in our room temperature experiments. Indeed, they must be caused by collisions between individual imaging electrons and individual target nuclei^11^. Therefore, one could expect that the transformations always occur when the electron probe is placed atop the defect, which would necessarily lead to detection of the transformation, as in Fig. 2e–h. Perhaps even more mysterious are the two frames where the defect has completely avoided detection (for an example, see Fig. 2i–k).

### Determination of the probe shape

**Figure 3:**
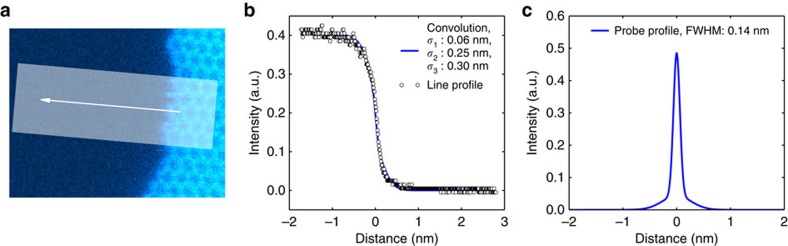
Determination of the probe shape. (**a**) Unprocessed (but coloured) image of a graphene edge. (**b**) Line profile obtained from the area shaded in panel **a** in the direction of the arrow along with a simulated line profile calculated assuming that the graphene edge is a step function and convoluting it with a probe that consists of three 2D Gaussians. The graphene edge position was set to the zero of the *x* axis. The s.d. for the Gaussians (*σ*_1_, *σ*_2_ and *σ*_3_) were obtained via manual fitting, with an estimated accuracy of ca. 10%. (**c**) One-dimensional profile of the probe consisting of the three Gaussians. Vacuum intensity was set to zero.

The answer to the transformation puzzle lies in the shape of the electron probe, which is pixel by pixel and line by line scanned over the area within the field of view (in our case 512 × 512 pixels within 4 × 4 nm^2^ for sequence 1 and 5 × 5 nm^2^ for sequence 2). While the full width at half maximum (FWHM) of the probe must be in the order of 1 Å for atomic resolution imaging, the actual shape of the probe and especially its tail^7^ further away from the point of the maximum intensity is not exactly known. In order to understand the role of the probe tails in our observations, we estimated the shape of our probe experimentally based on an intensity profile recorded over a graphene edge, as illustrated in Fig. 3. A good match between the recorded profile and a convolution of a step function with a model probe consisting of three 2D Gaussians (see Fig. 3b) was obtained for s.d. of *σ*_1_≈0.06 nm, *σ*_2_≈0.25 nm and *σ*_3_≈0.30 nm. The estimated accuracy of the manual fit is *ca*. 10%. A one-dimensional profile of the measured probe is presented in Fig. 3c. The FWHM for the probe is about 0.14 nm and the vacuum level is reached at a distance of about 1.5 nm. Less than 21% of the beam intensity is contained within the FWHM, which shows that a significant dose is deposited outside the beam maximum position. On the basis of this, and taking into account that the probe spends much more time at a distance of 0.07 nm <*r* ≤1.5 nm from the defect than on the defect itself (the middle-sized defect V_2_ (555–777) has an area roughly 5% of that of *π* (1.5 nm)^2^), it becomes clear that the effect of scanning near the defect can alter its atomic configuration. As noted, in our experiment this effect accounted for up to >85% of all of the transformations. When the transformations happen to drive the defect towards the already imaged area, this effect leads to its apparent disappearance for the duration of one or more scans.

### Statistical analysis of the random walks

**Figure 4:**
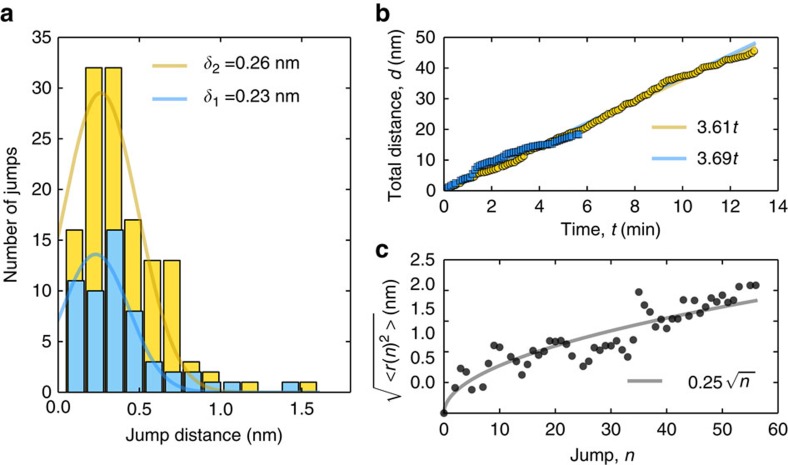
Statistical analysis of a random walk. (**a**) Histograms of all of the jump distances by the defect in the two image sequences. The lines show normal distributions fitted to the data. (**b**) Corresponding cumulative total distance travelled by the defect as a function of time. The lines are fits to the data. (**c**) Root-mean-square distance of the defect from the starting position as a function of time. The solid line is a fit to the data.

Histograms of all of the jumps between recorded frames in both of the image sequences are plotted in Fig. 4a. Assuming normal distribution, the average jump length is *δ*_1_≈0.23 nm for sequence 1 and *δ*_2_≈0.26 nm for sequence 2, which is close to the hexagon-hexagon distance in the graphene lattice. From the definition of diffusivity in two dimensions


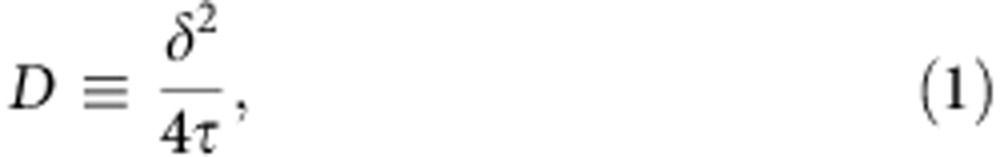


where 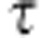 is the jump time, we get *D*≈3.1 × 10^−3^ nm^2^ s^−1^=3.1 × 10^−21^ m^2^ s^−1^ (using the value for the longer sequence), which is well in line with typical diffusivity values in solids. However, we stress that the migration is in the case of our experiment driven by the knock-on collisions between individual electrons and individual target nuclei, and the measured diffusivity is thus not directly comparable to values describing thermally driven point defect diffusion in solids.

Although some of the jumps between scans are considerably longer than others, the overall total cumulative distance (*d*) travelled by the defect increases linearly as a function of time (see Fig. 4b), yielding an estimated migration speed of 3.61 nm min^−1^ during the 13-min long experiment. For a random walk, the root-mean-square distance, which is a measure of the average distance of the walker from the start after *n* steps, is


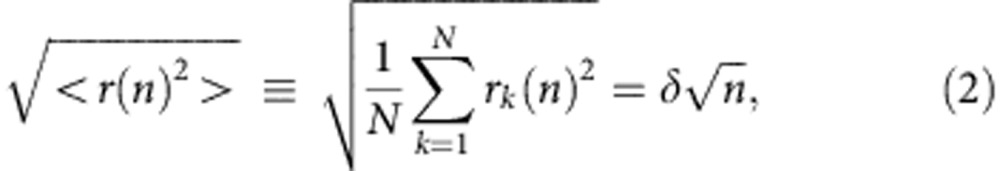


where *k* runs over the *N* different random walks (in our case the two image sequences) and *δ* is the average jump length. As can be seen in Fig. 4c, the random walks analysed here follow this behaviour. A fit to the data reveals *δ*≈0.25 nm, which is in between the above estimated values of *δ*_1_ and *δ*_2_, as can be expected.

We can take the analogue between thermal and electron-beam-driven diffusion one step further to compare our experimental observations with surface diffusion, where


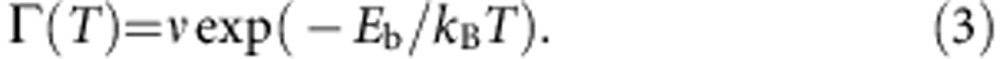


Here Γ(*T*) is the jump rate at temperature *T*, 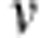 the attempt frequency, *E*_b_ the diffusion energy barrier and *k*_B_ the Boltzmann constant. For the area of the middle-sized divacancy, V_2_(555-777) and *E*_b_=5 eV as the energy barrier associated with a bond rotation^22^, we get an estimate for the conditions that we simulate with the electron beam. The apparent temperature of the system is *T*≈3,050 K, with the caveat that every recorded image is here assumed to represent exactly one migration step, whereas in reality we know that often several bond rotations have taken place between two subsequent frames. We stress that this is a virtual temperature, since the actual heat brought in by the electron beam is quickly dissipated away, and only modest if any actual heating of the sample is expected during the experiment^23^. Nevertheless, the observed defect migration is otherwise indistinquishable from what would be expected to occur at elevated temperatures: the energy input from the beam helps to overcome an activation barrier in a similar manner as heat would but—importantly—resulting in a process which we can directly image at room temperature.

## Discussion

As a conclusion, we have demonstrated for the first time that electron irradiation at 60 kV can be used to stimulate an atomic-scale random walk of a point defect in an otherwise pristine crystal, and to record it over several minutes. Despite the atomically small probe size, most of the transformations occur when the beam is situated away from the actual defect due to irradiation dose accumulation from the low-intensity tail of the electron beam. In rare cases, the defect can even completely avoid detection during a scan. Via analysis of the defect trajectory during the experiment, we estimate that the beam-stimulated migration of the divacancy corresponds to a virtual temperature of about 3,050 K, establishing atomic resolution transmission electron microscopy (TEM) as a method for simultaneous imaging and driving diffusion of point defects in low-dimensional materials.

## Methods

### Scanning TEM

The experiments were carried out with a Nion UltraSTEM 100 device^19^ recently installed at the University of Vienna. The device is equipped with a cold field emission gun, which was operated at 60 kV in ultra-high vacuum. Medium angle annular dark-field detector was used to record two image sequences of the same defect. The first sequence contains 57 frames recorded over about 5 min and 32 s with a field of view of 4 × 4 nm^2^. The second sequence contains 143 frames and was recorded over about 12 min and 59 s with a field of view of 5 × 5 nm^2^. Sample drift is less than the lattice spacing during the image sequences (the image sequences provided in Supplementary Movies 1–4 were not compensated for drift). The time the beam was held at each pixel was 16 μs for both sequences and all images contain 512 × 512 pixels. A typical camera current for the device is in the order of 5 × 10^−11^ A, from which a dose of circa 8 × 10^6^ *e*^−^ Å^−2^ was estimated per recorded frame for sequence 1 and 5 × 10^6^ *e*^−^ Å^−2^ for sequence 2.

### Image processing

The images were processed to reduce noise by applying a Gaussian filter with a radius of 6 px (for field of view of 4 × 4 nm^2^) and 4 px (for field of view of 5 × 5 nm^2^) after which the processed image was multiplied with the original image. The process was carried out twice for the second image sequence owing to lower signal-to-noise ratio.

### Determination of the defect position

To track the migration of the defect, we first aligned the image sequences, and then marked the middle of the defect manually to obtain the coordinates for each frame. The accuracy of this positioning procedure is estimated to be better than the interatomic separation in the lattice.

### Sample preparation

The graphene sample was grown via chemical vapour deposition and suspended on a TEM grid by a commercial supplier.

## Author contributions

J.K. initiated the study, carried out the experiment, analysed data and wrote the manuscript. C.M. contributed to establishing the experimental setup and J.C.M. contributed to the analysis. All authors commented on the manuscript.

## Additional information

**How to cite this article:** Kotakoski, J. *et al.* Imaging atomic-level random walk of a point defect in graphene. *Nat. Commun.* 5:3991 doi: 10.1038/ncomms4991 (2014).

## Supplementary Material

Supplementary Movie 1 A processed version of the first image sequence of the migrating divacancy defect (field of view 4 × 4 nm^2^).

Supplementary Movie 2 A processed version of the second image sequence of the migrating divacancy defect (field of view 5 × 5 nm^2^).

Supplementary Movie 3 A non-processed version of the first image sequence of the migrating divacancy defect (field of view 4 × 4 nm^2^).

Supplementary Movie 4 A non-processed version of the second image sequence of the migrating divacancy defect (field of view 5 × 5 nm^2^).
